# Current genetic differentiation of *Coffea canephora *Pierre ex A. Froehn in the Guineo-Congolian African zone: cumulative impact of ancient climatic changes and recent human activities

**DOI:** 10.1186/1471-2148-9-167

**Published:** 2009-07-16

**Authors:** Céline Gomez, Stéphane Dussert, Perla Hamon, Serge Hamon, Alexandre de Kochko, Valérie Poncet

**Affiliations:** 1 UMR DIAPC, IRD, 911 avenue Agropolis, BP 64501, 34394 Montpellier CEDEX 5, France

## Abstract

**Background:**

Among *Coffea *species, *C. canephora *has the widest natural distribution area in tropical African forests. It represents a good model for analyzing the geographical distribution of diversity in relation to locations proposed as part of the "refuge theory". In this study, we used both microsatellite (simple sequence repeat, SSR) and restriction fragment length polymorphism (RFLP) markers to investigate the genetic variation pattern of *C. canephora *in the Guineo-Congolean distribution zone.

**Results:**

Both markers were first compared in terms of their informativeness and efficiency in a study of genetic diversity and relationships among wild *C. canephora *genotypes. As expected, SSR markers were found to have a higher genetic distance detection capacity than RFLP. Nevertheless, similarity matrices showed significant correlations when Mantel's test was carried out (r = 0.66, p < 0.0001). Finally, both markers were equally effective for group discrimination and phylogenetic studies, but SSR markers tended to outperform RFLP markers in discriminating the source of an individual among diversity groups and in putative hybrid detection. Five well defined genetic groups, one in the Upper Guinean forests, the four others in the Lower Guinean forests, were identified, corresponding to geographical patterning in the individuals.

**Conclusion:**

Our data suggested that the Dahomey Gap, a biogeographical barrier, played a role in wild *C. canephora *differentiation. Climatic variations during the Pleistocene and/or Holocene probably caused the subgroup differentiation in the Congolese zone through the presence of a mosaic of putative refugia. Recent hybridization between *C. canephora *diversity groups, both for spontaneous individuals and cultivars, was further characterised according to their geographic dissemination or breeding history as a consequence of human activities.

## Background

The species richness in some African zones has attracted attention on the origin of diversification in tropical forests [1-4]. In particular, the Guineo-Congolean regional center of endemism (Zone I on White's map, 1983) includes about 8,000 spp., about 80% of which are endemic. This zone also corresponds to one of the 34 biodiversity hotspots defined by Mittermeier et al. [5]: the Guinean forests, divided into the Upper Guinean and Lower Guinean forests by the Dahomey Gap. Analyses of patterns of geographical distribution of species richness and endemism of extant organisms could partly help in understanding these species composition patterns [*e.g*. [3]]. Species richness in the tropics has been attributed to the gradual accumulation and/or preservation of species over a long geological period in stable equatorial climates (the "museum model") [6-8] and/or to high speciation rates in response to late Tertiary geological events and unstable Pleistocene climates [9,10]. In fact, all major glacial advances in the Arctic resulted in great paleoenvironment and vegetation variations in the African tropics [2]. At least for the most recently evolved taxa, a certain amount of speciation and a great deal of subspeciation is said to have occurred during the late Pleistocene climatic fluctuations, especially since the last severe glaciation which cumulated around 18,000 B.P. [11]. To explain how plants and animal species survived during particularly cold and arid periods, Maley [2] proposed putative refugia, where the respective ancestors of the present species formed isolated populations, leading to allopatric, or geographical speciation [12]. Some of the refugia were located along the West African coast from Sierra Leone to Côte d'Ivoire, from southwestern Cameroon to western Gabon, in the eastern part of the Democratic Republic of the Congo (formerly Zaire) and along the Zaire river [2]. Through a study of the African Rubiaceae genera distribution, Robbrecht [13] gave additional support in favour of the refuge area concept, and demonstrated the importance of fluvial refugia in some taxa.

Molecular phylogenies and species-level diversity could be used to further unravel the forest diversification patterns. A population-genetics rather than a species-level approach has also been proposed to test the role of the relatively recent time frame of Pleistocene events [14]. Climatic changes have affected the genetic structure of many tree species in Europe [*e.g*. [15-17]]. For Africa, major advances are under way for vertebrates [*e.g*. [14,18]], but very few studies have focused on tree species of West and Central Africa [19-22], and only one has attempted to establish the relationship between the molecular genetic variation of a tree species, *i.e*. the shea tree (*Vitellaria paradoxa*), and the possible evolution of vegetation after the last glaciations in the Sudano-Sahelian region of Africa [19].

*Coffea *species (Rubiaceae) are endemic to intertropical forest zones in Africa, Madagascar, Mauritius, Comoros and Réunion [23-27]. There are over 103 accepted species, including the two most commonly cultivated species, *C. arabica *L. and *C. canephora *Pierre ex A. Froehn [28]. Within *C. arabica*, a predominantly self-pollinating (autogamous) species, there is a genetic structure with low differentiation between accessions from the east and west side of the Great Rift Valley in Ethiopia as recently revealed with microsatellites [29]. The hybrid origin of *C. arabica *with *C. canephora *and *C. eugenioides *as the likely progenitors [30] was probably recent, and its colonization of Ethiopia likely occurred after the formation of the Great Rift Valley [31]. The systematic position and geographic distribution of *C. arabica *is isolated among diploid *Coffea *species [27,28]. On the contrary, *C. canephora *is one of the two *Coffea *species, along with *C. liberica *Hiern[28,32], with the widest natural distribution area of the genus. Moreover, these species are both diploid, allogamous (self-incompatible), and belong to the same phylogenetic clade [27]. They present overlapping geographical distributions, which extend west to east from Guinea to Uganda, and north to south from Cameroon to Angola [28,33]. This feature represents an exceptional resource for understanding the evolution and adaptation of tropical trees in these regions.

Few studies have been undertaken to assess the genetic diversity of natural *C. canephora *populations. Allozymic surveys revealed marked geographical clustering for two groups: the "Guinean" group, composed of populations from Côte d'Ivoire, and the "Congolese" group, consisting of two subgroups, SG1 and SG2, with populations from the Central African Republic and Cameroon [33,34]. Based on RFLP data, five wild *C. canephora *groups were further distinguished and the diversity structure was found to be conserved even when cultivated material, known as Robusta coffee, was included in the analysis [35]. Due to the increase in the number of microsatellite markers suitable for coffee species analyses [*e.g*. [36-38]], PCR-based assays are becoming increasingly attractive and compatible with the requirements of evolutionary studies or conservation genetics on large sets of genotypes.

Polymerase chain reaction (PCR)-based marker systems like simple sequence repeats (SSRs) have been widely used in recent years, replacing restriction fragment length polymorphism (RFLP) in DNA fingerprinting [39]. However, few studies have been carried out to compare the efficiency of RFLP and SSR markers for characterizing genetic diversity [40]. Both techniques offer the advantage of implementing nuclear co-dominant, locus-specific markers dispersed throughout the genome. Polymorphism detected by RFLP assays reflects restriction size variations, while microsatellite variants differ in the number of short (1–6 bp) amplified tandem repeats [41]. Their use in the assessment of population genetic structure has both advantages and drawbacks. While RFLPs assay are time consuming and labor intensive, among PCR-based markers, microsatellites require sequence information for primer pair design but, once developed, they are highly transferable across species, especially within the genus [*e.g*. [42,43]]. Because of their extremely high level of polymorphism, they are probably the most efficient markers for fingerprinting, assignment tests or paternity analyses [44]. Differences in the resolution power of RFLP and SSR loci are thus expected because of differences in their mutation rates and processes. The simultaneous use of these molecular methods could help in inferring the signature of natural histories of organisms at different time scales, *i.e*. evolutionary history and historic migration patterns. Moreover, the recent development of Bayesian approaches in assignment tests, as implemented in Structure[45], has greatly increased the potential for understanding population structure.

In the present study, we investigated *Coffea canephora *genetic diversity across the West and Central African geographic range of the species. Spontaneous individuals are naturally distributed in two postulated refugia areas, *i.e*. the Upper Guinea and Lower Guinea/Congo regions, separated by the Dahomey Gap. Using both SSRs and RFLP loci, we evaluated the relative efficiency of these DNA-based marker systems, associated with different mutation rates, in resolving genetic diversity, population structure and gene flow among samples of *C. canephora*.

The final aims were:

(i) to analyse the *C. canephora *genetic variability structure and assess any relationship between the molecular variation of the species and the possible signature of the past evolution of vegetation in the Guineo-Congolian region of Africa

(ii) to evaluate the impact of human agricultural activities on gene flow, dispersal and migration of both wild and cultivated stocks through the detection of putative inter-diversity group hybrids

(iii) to define core sets of *C. canephora *accessions that best capture the species diversity in terms of alleles.

## Results

### Efficiency of RFLP and SSR markers for polymorphism detection

**Figure 1:**
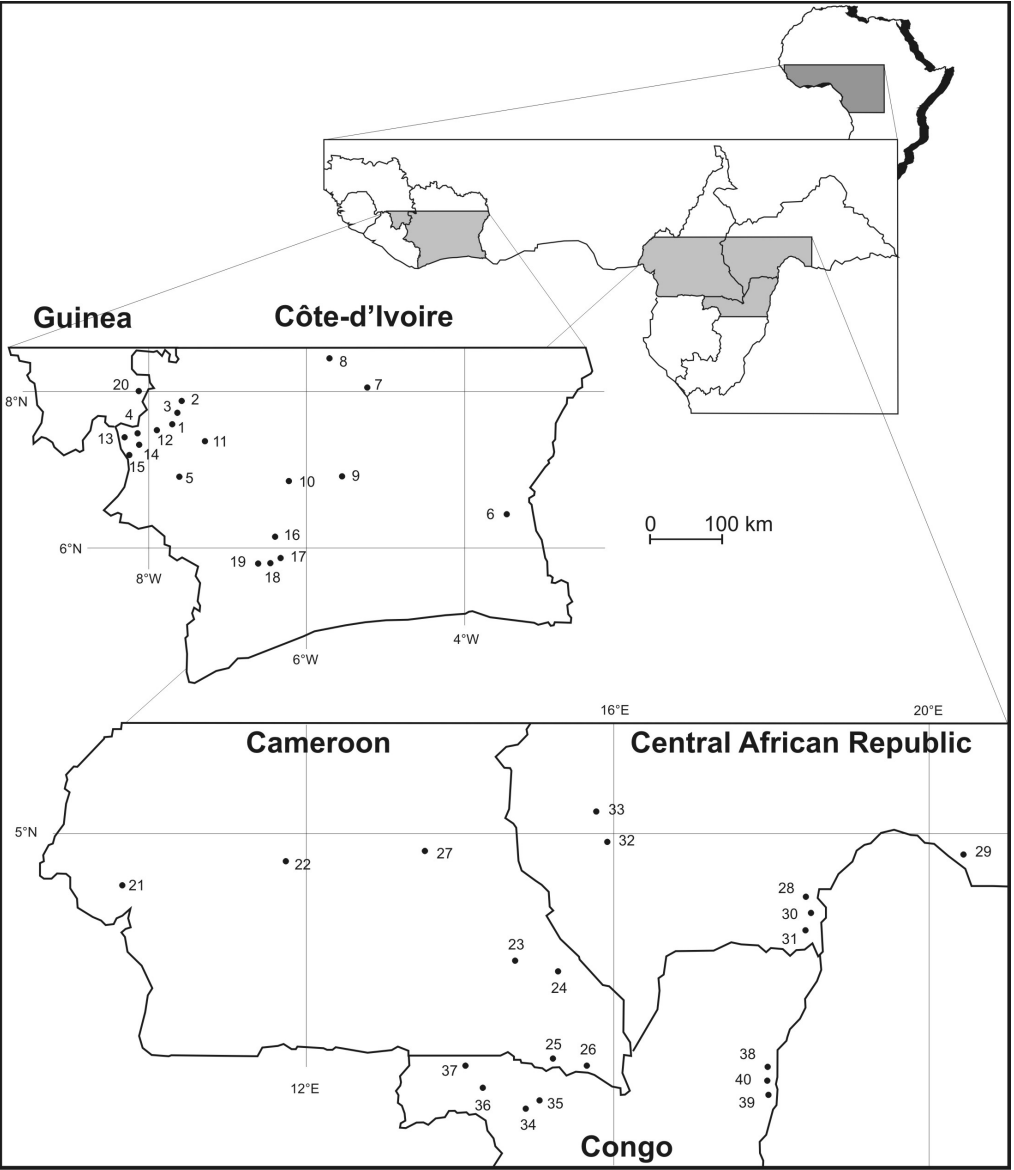
**Wild *Coffea canephora *sampling locations in West and Central Africa**. Codes are given in Table 1.

In the 107 sampled *Coffea canephora *accessions (Figure 1, table 1, and table 2), genetic polymorphism indexes (*Na*, *H*_O _and *H*_E_) at the 16 SSR loci and 8 RFLP loci were found to be highly variable throughout all geographic samples and types (Table 3). A total of 154 alleles across the SSR loci were detected, giving an average of 9.6 alleles per locus, ranging from 3 at M804 to 20 at M368. In comparison, the RFLP loci analysed gave 59 alleles with an average of 7.4 alleles per locus. The PIC value and gene diversity were quite even for SSR and RFLP loci, *i.e*. 0.62 *vs*. 0.59 and 0.65 *vs*. 0.63, respectively.

**Table 1:** Geographic and genetic origin of the wild *C. canephora *genotypes

	Location					*A priori *genetic	Putative
Country	#	Name	Long.	Latit.	Coll. #	group	hybrids
Côte d'Ivoire^1,2^	1	Ira 1	07.40	-07.46	0009 0028	D	
	
	2	Bafingdala	07.51	-07.41	0056	D	
	
	3	Ira 2	07.50	-07.42	0105	D	
					0159	D	
	
	4	Gbapleu	07.34	-08.18	0121	D	
					0128	D	
	
	5	Gao	06.57	-07.39	0136	D	
	
	6	Bossematie	06.30	-03.30	0145	D	
					0146	D	
	
	7	Logbonou	08.04	-05.15	0186	D	DA
					0194	D	DE
	
	8	Fourougbankoro	08.29	-05.45	0213	D	
					0233	D	
	
	9	Goazra	07.00	-05.36	0236	D	
	
	10	Marahoué	06.54	-06.12	0245	D	
					0250	D	
	
	11	Kouin	07.30	-07.18	0292	D	
					0293	E	
	
	12	Géoulé	07.36	-07.56	0313	D	
					0315	E	
	
	13	Gbapleu 1			0319	E	
					0321	E	
	
	14	Gbapleu 2			0328	D	
	
	15	Gbapleu 3			0336	D	
	
	16	Dobia			0345	D	DEA
					0350	D	DE
	
	17	Sabrégué			0354	D	
					0358	D	DE
	
	18	Pélézi 2			0362	D	
					0395	D	
	
	19	Gonaté			0404	D	

Guinea^2^	20	Piné	08.57	-08.06	0803	D	
	20	Piné	08.57	-08.06	0808	D	

Cameroon^3^	21	Koto	04.22	09.34	0651	E	
	
	22	Nguila	04.43	11.40	0658	C	DA
	
	23	Mwamepen	03.29	14.48	0662	E	
					0663	A	AE
	
	24	Bitonga	03.19	15.16	0664	C	
	
	25	Boumba	02.03	15.10	0665	E	
					0666	E	
	
	26	Nguilili	02.04	15.36	0678	C	
					0683	C	
	
	27	Bombi	04.56	13.10	0685	C	

Central African Republic^4^	28	Loukoussou			0501	B	
	
	29	Doungba			0504	E	
					0511	B	
	
	30	Yombi			0516	E	
	
	31	Libengué			0518	B	
	
	32	Carnot			0602	C	
					0604	C	CE
	
	33	Ndongué			9004	C	

Congo^5^	34	Ouesso 2	01.35	14.48	0716	C	
	
	35	Ouesso	01.37	14.53	0721	C	
	
	36	Sembé-Souanké	01.56	14.11	0723	A	
	
	37	Souanké-gabon	02.07	14.00	0725	E	
					0727	E	
	
	38	Boyélé 5			0729	E	
					0730	E	
	
	39	Impfondo 1			0738	E	
	
	40	Impfondo 2			0739	E	
					0740	E	

Membership to genetic groups, as previously defined by Dussert et al. [35] and putative hybrids detected in the present study are indicated for each accession.

^1^Berthaud [33]; ^2^Le Pierrès et al. [26]; ^3^Anthony et al. [24]; ^4^Berthaud and Guillaumet [23]; ^5^de Namur et al. [25]

**Table 2:** Geographic and genetic origin of the cultivated *C. canephora *genotypes.

Type of introduction	Group name	Donor or collector	Country of origin	Coll #	*A priori *genetic group	Final group
Donation from field genebanks	"Aboisso"	Aboisso^1^,	Gabon	C135	A	A
		Côte d'Ivoire		C307	A	A
				C318	E	DE
				C329	E	AE
	
	"C10 Man"	Unknown	Rep. of Congo	C429	E	E
				C439	E	E
	
	"INEAC"	INEAC^4^,	Rep. of Congo	C002	E	E
		Rep. of Congo		C078	E	E
				C032	E	E
				C054	E	E
				C057	E	AE
				C062	E	E
				C095	E	DE
				C003	A	AE
				C006	E	E
				C015	E	E
	
	"Kouilou of Madagascar"	Bingerville^3^, Côte d'Ivoire	Gabon	CK07	A	A
				CK14	A	A
				CK29	A	AE
	
	"Niaouli"	Bingerville^2^, Côte d'Ivoire	Togo	C008	A	A
				C017	A	A

Collection in plantations	"Guinea"		Guinea^6^	0855	D	D-
				0856	D	DE
				0850	E	DE
				0851	E	DE
				0852	E	E
				0909	E	DE
				0915	E	DE
				0848	E	DE
				0853	E	D-
				0881	E	DE
				0882	E	DE
	
	"Hybrid"		Côte d'Ivoire	C107	D	DE
				C126	E	DE
				C181	D	DE
				C636	D	DE
	
	"Côte d'Ivoire"		Côte d'Ivoire^5,6^	0317	D	DE
				0318	D	DE
				0163	A	AE
				0164	A	A
				C155	A	D
	
	"Togo"		Togo	0693	A	A
				0695	A	A
	
	"Tanzania"		Tanzania	0270	Unknown	E-
				0279	E	CE

Unkown	"Robusta A1"		Unknown	C077	A	A

Membership to *a priori *genetic groups, as previously defined by Dussert et al. [35], and final group definition as identified in the present study are indicated.

^1 ^Introduction at Aboisso (Côte d'Ivoire) by Beynis in 1910, of material cultivated in Gabon [57]

^2 ^Introduction at the garden at Bingerville (Côte d'Ivoire) in 1914 of material cultivated in Togo [57]

^3 ^Introduction at CRA Bingerville (Côte d'Ivoire) in 1951, of material selected in Madagascar and originating from Gabon [57]

^4 ^Introduction in Côte d'Ivoire in 1935 of material selected at INEAC in the Repubic of Congo (Cordier [57])

^5^Le Pierrès et al. [26]; ^6^Berthaud [33]

**Table 3:** Characteristics of the 16 SSRs and 8 RFLP loci from the total sample (N = 107) and the wild sample (N = 61).

SSR									
	Map location	Total sample N = 107				Wild sample N = 61			
		
Locus	LG^1^	*N*_a_	*H*_O_	*H*_E_	PIC	*N*_a_	*H*_O_	*H*_E_	PIC
		
M257	B	6	0.25	0.55	0.47	6	0.21	0.59	0.52
M809	H	5	0.15	0.25	0.23	4	0.11	0.16	0.15
M804	M	3	0.06	0.07	0.07	1	0.00	0.00	0.00
M821	F	13	0.28	0.83	0.81	13	0.28	0.83	0.81
M314	G	5	0.25	0.49	0.43	5	0.18	0.42	0.36
M368	-	20	0.81	0.93	0.92	15	0.76	0.91	0.91
M764	-	13	0.65	0.83	0.81	11	0.63	0.82	0.80
M394	C	9	0.41	0.80	0.77	8	0.39	0.75	0.71
M779	-	12	0.75	0.85	0.83	12	0.63	0.82	0.80
M782	-	4	0.06	0.25	0.22	4	0.03	0.12	0.11
M495	-	9	0.35	0.65	0.60	9	0.33	0.62	0.57
M259	N	6	0.52	0.59	0.55	6	0.48	0.60	0.56
M367	-	15	0.30	0.88	0.87	12	0.20	0.85	0.83
M755	-	13	0.42	0.78	0.76	11	0.23	0.68	0.65
M387	J	10	0.33	0.75	0.71	8	0.26	0.74	0.70
M856	I	11	0.49	0.88	0.87	11	0.41	0.85	0.84
									
**Total**		**154**				**136**			
Mean		9.63	0.38	0.65	0.62	8.5	0.32	0.61	0.58
RFLP									
		N = 107				N = 61			
		
Locus	LG^2^	*N*_a_	*H*_O_	*H*_E_	PIC	*N*_a_	*H*_O_	*H*_E_	PIC
		
gA13	10	14	0.51	0.73	0.70	11	0.43	0.66	0.61
gA19	9	10	0.43	0.78	0.75	9	0.38	0.72	0.68
gA29	5	5	0.27	0.47	0.44	5	0.26	0.50	0.47
gA10	3	8	0.48	0.78	0.76	7	0.42	0.77	0.74
gA14	3	5	0.30	0.59	0.53	5	0.31	0.56	0.52
gA61	7	8	0.50	0.70	0.66	6	0.48	0.69	0.65
cR167	4	7	0.43	0.60	0.57	7	0.34	0.58	0.55
gA72	7	2	0.16	0.41	0.33	2	0.13	0.34	0.28
									
**Total**		**59**				**52**			
Mean		7.37	0.38	0.63	0.59	6.50	0.34	0.60	0.56

Linkage group locations for mapped markers are given. The number of alleles (*N*_a_), observed heterozygosity (*H*_O_), expected heterozygosity (*H*_E_, or gene diversity) and PIC (polymorphism information content) values are also given.

^1 ^Linkage group of (*C. canephora *× *C. heterocalyx*) × *C. canephora *genetic map from Coulibaly et al. [60]

^2 ^Linkage group of *C. canephora *genetic map from Paillard et al. [59], with estimated distances: gA10-gA14 = 21 cM, gA61-gA72 = 30 cM

### Structure of *C. canephora *natural diversity

Dissimilarity matrices were constructed on the whole sample based on shared-allele distances and revealed that the average genetic dissimilarities for RFLP (0.604) and SSR (0.626) markers were very similar. The average genetic dissimilarities obtained for the wild genotypes were also similar for both marker types, RFLPs (0.581) and SSRs (0.595). The correlation coefficient obtained with Mantel's test matrix correspondence test was, indeed, statistically significant (r = 0.66, p < 0.0001).

**Figure 2:**
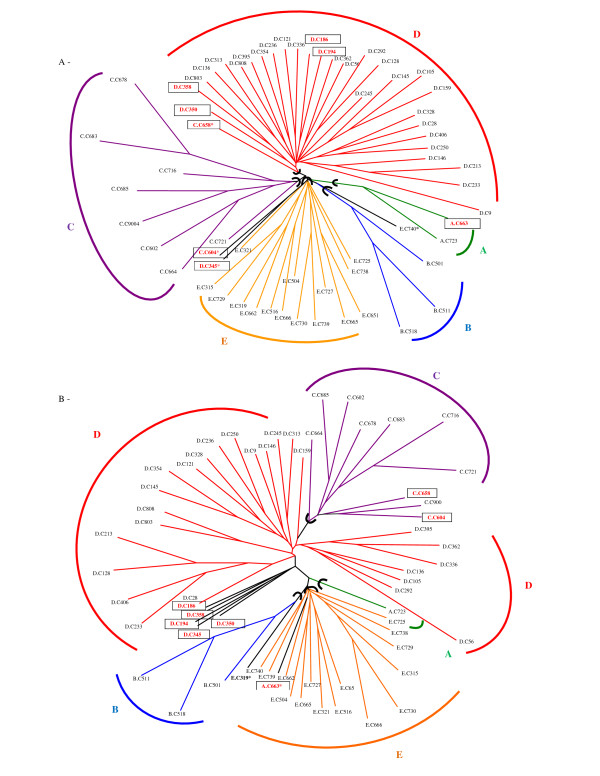
**Unrooted trees of individual wild *Coffea canephora *genotypes using the neighbour-joining method and shared-allele distance among (A) 16 microsatellite loci and (B) 8 RFLP loci**. The first letter of the individual labels, A, B, C, D and E, indicates the a *priori *diversity group of Dussert et al. [35]. Individuals who appear to be misclassified in the present tree are marked (*). Circled names represent plants identified as being miss-classified or putative hybrids by the subsequent genetic structure analysis.

Genetic distance estimates obtained from the wild genotypes were used to derive Neighbour-joining trees for both the RFLP and SSR data. The topology of each tree (Figure 2) was unique but both trees discriminated the five *a priori *genetic groups (A, B, C, D, and E) from Dussert et al. [35]. Nevertheless, the SSR tree was not completely congruent with the RFLP tree. One difference was that group C, which occupied an intermediate position on the tree based on SSRs, occurred at a more distant position from groups [B-E-A] within group D based on RFLPs. Another contrast was noted with respect to the A individuals which clustered together near the E group based on SSRs and one of its individuals fell into the group E, based on RFLPs. The internal branches were generally longer for the RFLP tree. Despite these differences, both tree topologies reflected the same distinct clades, corresponding to a geographical patterning in the individuals. Plants from Côte d'Ivoire mostly clustered with plants from Guinea (group D). Individuals from northwest Congo, southeast Cameroon, and southwest Central African Republic fell within the same clade (group C), in line with their geographic proximities in Central Africa. The plants of Central African group C clustered near plants from West Africa (group D). From the eastern part of the geographical distribution, plants from northeast Congo clustered with plants from southeast Cameroon and south Central African Republic (group E), near plants from the southern border of Central African Republic (group B). The genotypes of a population from northwest Congo and a population from southwest Cameroon (group A) clustered near the B and E clades. Several individuals (marked with asterisks) appeared to be classified with groups other than their *a priori *groups. We performed a population structure analysis to further assess the group memberships of the plants and to detect actual migrants, hybrids or misclassified individuals.

**Figure 3:**
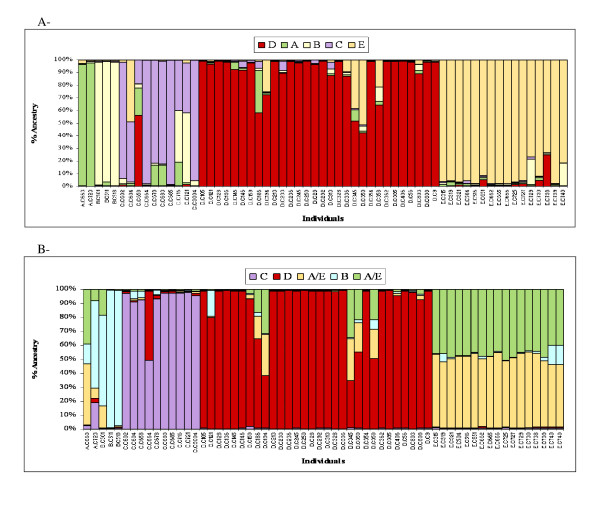
**The genetic structure of wild *Coffea canephora *(N = 61) based on (A) 16 polymorphic SSR loci (B) 8 polymorphic RFLP loci**. Result of Structure analysis using K = 5 and assignment to the five *a priori *diversity groups (A to D) from Dussert et al. [35]. The colour bars represent percentages of ancestry from the different groups observed in one individual.

The structure analysis using Structure with admixture showed that the five groups (K = 5) were genetically distinct based on SSR data (Figure 3). The results indicated that most of the plants had a high membership in their own cluster, with 97, 97, 69, 90, and 92% mean ancestry for the A, B, C, D, and E groups, respectively. In comparison, with the RFLP data, plants from groups B, C, and D formed separated clusters with high membership in their own cluster (87, 91 and 88% on average, respectively). However, mixed ancestry between groups A and E at even proportions (A and E contributing to 50 and 44% ancestry on average) was observed, corresponding to a grouping by geographical regions. For this mixed group, we considered that plants possessing <80% ancestry in the [A+E] cluster were putative hybrids. Despite this discrepancy, the level of admixture per group was very low and similarly estimated by both markers (Table 4). Eight genotypes were identified as admixed by SSRs, with over 20% ancestry from other groups. Five of them were also identified by RFLPs. Some of these putative hybrids, 4/7 and 2/5 detected by SSRs and RFLPS, respectively, were confirmed with the reassignment test implemented in Geneclass2 (Table 4). The five individuals identified as putative hybrids by structure analysis using either marker type originated from the *a priori *D group of Côte d'Ivoire, and contained >20% group E or group A ancestry (Table 4). On the RFLP tree, these individuals nested within the same clade, with an intermediate position on the between groups [A-B-E] and [C-D]. They occurred at the base of the D clade on the SSR tree. The individual AC663, classified *a priori *in group A, and identified as a putative AE hybrid with RFLPs, fell into the group E cluster in the RFLP tree.

**Table 4:** Missclassified wild individuals or putative hybrids detected by Geneclass and Structure analyses.

	*A priori *Genetic Group	Geneclass			Structure		
			
		RFLP	SSR	RFLP		SSR	
Individuals				Ancestry (%)	CI	Ancestry (%)	CI
			
AC663	A	E 1.445		E 43.5 A 39 B 14.2	0–100 0–100 0–61		
CC604	C	-	E 3.224		-	E 49.1 C 47.4	23.3–76.2 18.4–73.5
CC658	C	-	D 5.010		-	D 56.3 A 21.2	24.8–83.6 0–71.9
DC186	D	-	-	D 64 E 16 A 16	36.1–86.5 0–52.8 0–54.2	D 57.9 A 33.5	29.6–84 0–68.3
DC194	D	-	-	D 37.5 E 29.6 A 31.6	0–66.1 0–85.8 0–94.2	D 72.6 E 25.3	48.3–94.4 0–50.3
DC345	D	A 0.579	E 0.526	D 33.7 E 30 A 34.3	0–65.2 0–97.3 0–997	D 51.5 E 38.7	26.7–74.7 12.9–65.2
DC350	D	-	E 1.553	D 54.5 E 20.7 A 21.7	0–87.1 0–75.5 0–91.7	D 42.4 E 51.6	19.8–65.5 14.2–76.9
DC358	D	-	-	D 49.9 E 21 A 21.5	12.1–77.9 0–66.6 0–68.5	D 64.3 E 21.2	42.6–83.7 0–51.2

The alternative group and log10 L_home/L_max from the Geneclass analysis is given. The Structure results are given for the percentage of admixture (mean ancestry) of each of the five diversity groups and the 90% CI. Cells are kept empty for individual non-detected hybrids, *i.e*. over 80% membership to their own cluster.

The genetic differentiation of wild *C. canephora *groups, evaluated with *F*_ST _values, were found to be higher for RFLPs compared to SSRs, with 0.50 *vs*. 0.33, respectively (Table 5). Pairwise RFLP-based or SSR-based *F*_ST _values ranged from 0.29 to 0.67 and from 0.20 to 0.50, respectively, with substantially lower *F*_ST _values within regions than between regions. Group D (West Africa) showed the highest levels of differentiation detected with all other groups with both marker types, whereas the A-B and E-B comparisons (groups from Central Africa), gave the lowest *F*_ST _values with RFLP and SSR data, respectively. Mantel's tests revealed that pairwise *F*_ST _estimates among samples calculated from SSRs were not significantly different from those calculated from RFLPs (R = 0.72, N = 1000 permutations).

**Table 5:** Pairwise *F*_ST _estimates of genetic divergence in wild *C. canephora *groups (N = 53) obtained with SSRs (below the diagonal) and RFLPs (above the diagonal)

SSR\RFLP	A	B	C	D	E
A	0	0.29	0.50	0.67	0.30
B	0.33	0	0.51	0.67	0.30
C	0.30	0.24	0	0.54	0.41
D	0.50	0.45	0.32	0	0.52
E	0.31	0.20	0.25	0.34	0

### Cultivated sample origin

When the cultivated genotypes were included in the neighbour-joining tree construction, the topology of the trees in five groups could not be identified appropriately with RFLPs and the clusters were even less separated using SSRs (data not shown). Since some of the cultivated plants might be of putative hybrid origin (Table 2, "hybrid" group), we assessed the cultivated genotype origin by population structure analysis.

Assignment tests using Geneclass were first performed to assess genotypic similarities of the cultivated plants with respect to the five wild genetic reference groups. Using RFLPs, of the 46 cultivated plants, 10 (22%), 13 (28%) and one (2%) were assigned to groups A, E, and D, respectively, whereas the remaining individuals (48%) were all unassigned. In comparison, SSRs only assigned 8 (17%), 9 (20%) and one (2%) individuals to groups A, E, and D, respectively, whereas 28 (61%) remained unassigned. Considering the overall dataset, assigned cultivated individuals, except for three of them, were correctly classified within their *a priori *group of origin with both RFLP and SSR data. Genetic groups B and C were not represented by cultivated genotypes. None of the *a priori *group D individuals were reassigned to D, suggesting a possible mixed ancestry origin.

Genetic admixture analysis was conducted with Structure using the overall dataset with both cultivated and wild plants, while excluding wild individuals from group B. The four groups (K = 4) observed, corresponding to groups A, C, D, and E, were found to be genetically distinct with either RFLPs or SSRs. The mean proportion of population membership of wild genotypes to their own clusters was 72, 84, 90, and 92%, for groups A, C, D and E, respectively, using RFLPs; and 69, 82, 89, and 93% for groups A, C, D and E, respectively, using SSRs. This confirmed that the previously defined genetic groups were still responsible for the observed population structure, even after the addition of cultivated individuals.

Admixture analysis with RFLPs of individual genotypes identified hybrids among individuals from *a priori *group A (3/14 = 21%) and *a priori *group E (8/24 = 33%). All plants from *a priori *group D were detected as hybrids between groups D and E. In comparison, the SSR analysis detected slightly more hybrids with 4/14 = 29% in *a priori *group A, 13/24 = 54% in *a priori *group E, and all plants from *a priori *group D.

Most of the hybrids 13/17 = 76% and 13/23 = 57%, for RFLPs and SSRs, respectively, were identified as originating from hybridization between *a priori *groups D (West Africa) and E (Central Africa). These hybrids shared an equivalent average fraction of ancestry from both groups (49%D-45%E with RFLPs and 43%D-48%E with SSRs), suggesting that most of them could be classified as first generation hybrids.

The combined data analysis of cultivated coffee trees using both marker types and both population structure analyses allowed us to identify the putative hybrid origin of many cultivated plants (25/46 = 54%) (Table 2).

**Figure 4:**
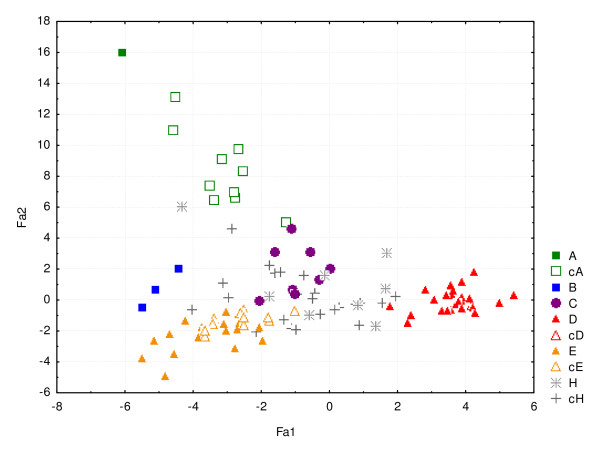
**Principal component analysis of wild and cultivated *C. canephora *accessions based on their SSR polymorphism**. The first axis represents 10% of the variation and the second one represents 6.5% of the variation. The wild *C. canephora *accessions are represented by colored symbols (A, B, C, D, E). The cultivated accessions assigned to wild genetic groups are represented by empty corresponding symbols (cA, cD, cE). The individuals identified as putative hybrids are represented by a cross mark, H for the wild accession and cH for the cultivated ones.

Principal coordinate plots representing the genetic similarity between wild and cultivated *C. canephora *for SSRs are presented in Figure 4 on the basis of SSR data. The variance explained by the first two axes was greater for RFLPs than for SSRs (45.2% *vs*. 16.7%). However, on both plots, wild individuals were genetically distinct, with no overlap of the five genetic groups. The distribution of cultivated individuals overlapped that of wild groups, with putative hybrids located at intermediate positions.

### Core set selection

We selected core sets of both wild and cultivated accessions from *C. canephora *that capture the maximum number of SSR or RFLP alleles for sample sizes 12 and 25. Core sets of 12 captured 48/59 and 103/154 of the RFLP and SSR alleles, respectively. Gene diversity in each core set was equivalent to that found in the entire sample (Table 6). Each core set contained wild and cultivated accessions from the different groups, and a substantial fraction of putative hybrids.

**Table 6:** Core set of 12 or 25 *C. canephora *genotypes maximising RFLP and SSR diversity.

RFLP	Total:	*N*_a _= 59	SSR	Total:	*N*_a _= 154
		GD107 = 0.63			GD107 = 0.65
	
Core set of 12	Statut	Core set of 25	Core set of 12	Statut	Core set of 25
	
_C270	(c) H	_C270	BC511	(+) B	BC511
DCC181	(c) H	DCC181	ECC002	(c) E	ECC002
ECC057	(c) H	ECC057	ECC429	(c) E	ECC429
ECC439	(c) E	ECC439	EC279	(c) H	EC279
CC9004	(+) C	CC9004	ACC135	(c) A	ACC135
EC853	(c) H	EC853	BC501	(+) B	BC501
CC721	(+) C	CC721	_C270	(c) H	_C270
AC164	(c) A	AC164	CC664	(+) C	CC664
EC315	(+) E	EC315	DC9	(+) D	DC9
BC501	(+) B	BC501	ECC095	(c) H	ECC095
EC319	(+) E	EC319	EC851	(c) H	EC851
BC518	(+) B	BC518	DC146	(+) D	DC146
	(c) H	EC848		(+) C	CC716
	(+) E	EC666		(c) A	ACC008
	(c) H	EC915		(+) H	CC658
	(+) H	CC658		(+) H	DC186
	(c) H	EC881		(+) E	EC739
	(+) E	EC651		(c) H	ECC057
	(+) D	DC803		(c) H	EC882
	(+) C	CC716		(+) D	DC136
	(c) H	EC850		(c) A	ACK07
	(c) H	EC882		(c) H	ACK29
	(c) E	ECC429		(+) H	CC604
	(+) E	EC740		(c) A	AC164
	(c) H	EC279		(+) H	AC663
	
*N*_a _= 48		*N*_a _= 58	*N*_a _= 103		*N*_a _= 137
36.9%		59.6%	19.6%		38.3%
GD = 0.68		GD = 0.72	GD = 0.66		GD = 0.69

Corresponding gene diversity (GD) values, cultivated status: wild (+) or cultivated (c), genetic group (A to E) or putative hybrid (H), No. of alleles (*N*_a_) and percentage of total variability revealed in the whole sample (107 individuals) are given.

## Discussion

Our analysis of *C. canephora *genetic diversity through RFLP and SSR markers revealed a marked geographic structure in subgroups, which could be interpreted with a broad scope of regional and historical influences. The detection of inter-diversity group putative hybrids allowed us to evaluate the influence of humankind on the original natural distribution and the occurrence of gene flow between wild and cultivated stocks.

The choice of the appropriate marker for any specific study depends principally on the purpose of the research and the biology and genetic structure of the species. The recent development of assignment tests based on Bayesian approaches [45-47] has greatly increased the potential for understanding population structure across its diversity range. In parallel to our SSR study, we thus re-analysed the RFLP data from Dussert et al. [35] on the same accessions to better compare and assess the associated diversity.

### RFLP – SSR comparison

#### Direct marker system comparison

The genetic data parameters obtained in this study from SSRs were first compared with those obtained with RFLPs.

One of the greatest differences between the two categories of markers concerns their respective polymorphism levels. Estimates of genetic variability *H*_*E *_and PIC-values, were higher for SSRs than those calculated from RFLPs. This difference in allele variation reflects differences in mutation level: replication slippage is indeed thought to occur more frequently than single nucleotide mutations and insertion/deletion events. RFLP loci are thus characterized by lower mutation rates as compared to SSRs, whose mutation rates can range from 10^-3 ^to 10^-4 ^depending on their genomic position [48]. Levels of polymorphism detected with RFLP and SSR markers have been compared in soybean [40], also indicating the hypervariability of SSR loci and greater expected heterozygosity.

The higher mutation rates of SSRs also had an effect on genetic distance values. The dissimilarity values taken over all pairwise comparisons were on average higher for SSRs (0.63) than for RFLPs (0.60). However, the comparison of both dissimilarity matrices revealed that estimates based on RFLPs and SSRs were highly correlated (r = 0.66, p < 0.0001), indicating congruence between the assays. As a consequence of the mutation rates, the PCO gave a higher percentage of variation explained by the two first axes with RFLPs than for SSRs (45.2% *vs*. 16.7%), indicating a better separation of groups with fewer multilocus genotypes. However, both plots gave similar resolution in the distinction between individuals of the different groups. Our two NJ trees mainly gave congruent results.

#### Discriminatory power and assignment capacity

The overall information generated by both markers to facilitate the determination of phylogenetic relationships and classifications, cluster analysis and population structure analysis in the *C. canephora *gene pool was further assessed. Indeed, the differences in mutation rates would also likely affect the power of the different markers to detect population differentiation.

In our data, overall and pairwise *F*_ST _estimates obtained with RFLPs were higher than those obtained with SSRs. However, in spite of the differences, SSR and RFLP multilocus *F*_ST _estimates were not significantly different (R = 0.72) when computed over the entire set of samples. In fact, the high level of polymorphism, typical of SSRs, may induce downward bias in the population differentiation estimates. The degree of differentiation assessed through SSRs, and thus the *F*_ST _values, are expected to be lower than those calculated using RFLPs [49]. A marked population subdivision similar to that noted in our study was also found at RFLP loci in brown trout [50] and soybean [40]. Moreover, the consistency of our results obtained from both markers is congruent with the findings of the other study comparing SSR and RFLP variation [40].

Finally, the RFLP and SSR multilocus diversity structure analysis divided wild coffee trees into largely concurrent five groups, with main branches on the individual neighbour-joining tree. Comparable diversity structuring was obtained, *e.g*. in terms of distinguishing geographical origins from West Africa and Central Africa.

At a smaller geographical scale, RFLPs and SSRs showed a different resolution power in detecting the genetic structure in the wild samples. The Bayesian analysis using Structure revealed five clearly distinct groups with SSR data, while samples from the southwest Cameroon/northwest Congo region–*a priori *groups A and E–were clustered with the RFLP data. A higher membership in their own cluster was obtained with SSRs (89% *vs*. 72% mean ancestry over all wild groups with SSRs and RFLPs, respectively). Moreover, the SSR data allowed the identification of slightly more admixed plants, putatively hybrids, both within the wild and cultivated pools. The Geneclass assignment test, which has been shown to be effective even if clusters are not in HWE, gave congruent results.

In conclusion, SSR markers outperformed RFLP markers in terms of discriminatory power in cluster analyses and assignment tests. Moreover, SSRs were also more efficient in discriminating the source of an individual genotype among putative diversity groups on a local scale. This advantage of SSR markers when analysing genetic affinities at individual levels was also demonstrated in a study comparing SSR and allozyme markers in brown trout [50].

### *C. canephora *genetic diversity and history

#### Organisation of *C. canephora *natural diversity

The genetic structure analysis carried out on the African samples of wild *C. canephora *using RFLPs and SSRs revealed marked separation between the West and Central African samples corresponding, in their composition, to the Guinean and Congolese groups of Berthaud [33]. This marked separation might be related both to the large geographical distances and to historical events. Indeed, the last glaciations and the subsequent migration from various Pleistocene refugia had caused large-scale changes in vegetation patterns, most notably around the Dahomey Gap and Cross River [2]. The western forests from Guinea and Côte d'Ivoire are separated from the Central forests of Cameroon, Central African Republic and Congo by the current 300 km wide Dahomey Gap, which is known to be an important biogeographic barrier. This area is thought to have become an extremely arid and much wider area during the last glacial maxima, around 18,000 BP, separating the forest refuges of southwest Ghana and west Cameroon [2]. Divergence through this geographical isolation might have led to genetic differentiation of the *C. canephora *populations. When analysing *C. liberica*, another *Coffea *species with the same geographical distribution, N'Diaye et al. [32] reported that the two varieties *C. liberica *var. liberica Bull. ex Hiern and *C. liberica *var. dewevrei (De Wild. & T. Durand) Lebrun, had high genetic differentiation and were characterised by marked reproductive barriers between the two varieties, with a pollen viability of their F_1 _hybrids similar to that of inter-specific hybrids. However, although highly differentiated, the West (group D) and Central African (A, B, C, and E) groups of *C. canephora *present fully interfertile individuals (see below). This suggested that the two main *C. canephora *diversity groups do not present a state in the speciation process as advanced as that of the two *C. liberica *varieties. Phylogenetic studies have indeed revealed the role of the Dahomey Gap on lineage origins in the Upper Guinea regions [27,51]. But a similar distinct evolutionary split between populations in Upper Guinea and those of Lower Guinea across the Dahomey Gap has also been observed in shea tree species [19] and in Fire-crested Alethe birds [52].

**Figure 5:**
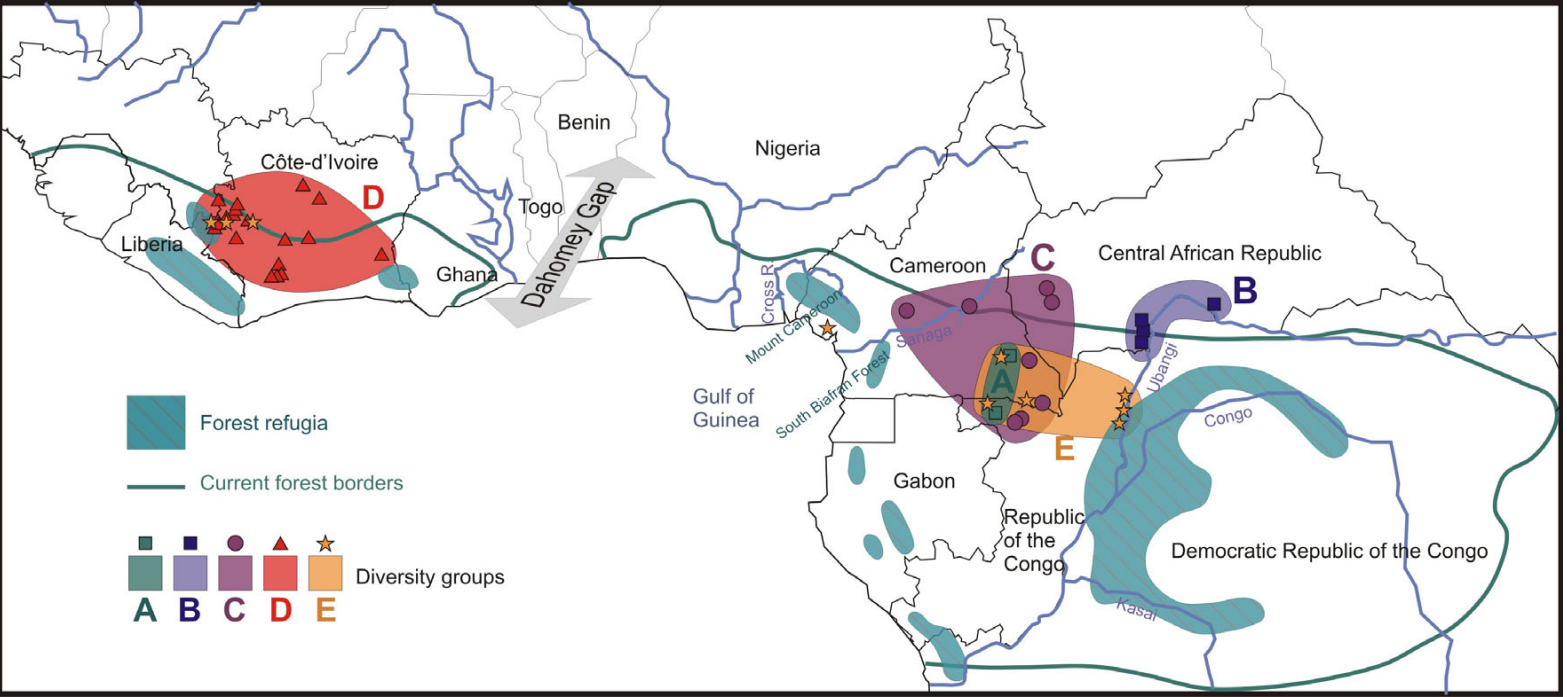
**Geographic position of the five major *C. canephora *genetic subgroups**. Geographic position of the five major *C. canephora *genetic subgroups collected together with a schematic map of forest refugia over the course of the last major arid phase (c. 18,000 years BP), adapted from Maley [2].

The refugia scenario concerning the African Guineo-Congolian rainforest is supported by the fact that a similar speciation pattern has been observed in various genera [reviewed in [4]], particularly in African Rubiaceae genera [13]. Isolation of populations in refugia was also suggested to influence divergent adaptations and diversification at the species level [*e.g*. [14,18]]. Within the Central African zone, the distribution of the four *C. canephora *groups (A, B, C, and E) showed a complex pattern. This regional pattern of differentiation could be interpreted as evidence of cycles of fragmentation and subsequent expansion of forest habitats. The Congolese region consisted of a mosaic of several Pleistocene refugia [2] that might explain the pattern of genetic diversity in the Central African zone. Although it is difficult to precisely localise the refugia, the reconstructed hypothesized areas [2] were found to be related to species diversification. For example, Anthony et al. [14] suggested a role of these Pleistocene refugia in structuring gorilla genetic diversity. For *C. canephora*, the refugia origin of sub-group E is most likely located in the Congo-Zaïre basin (Figure 5). Meanwhile, the *C. canephora *group C might derive from the expansion of the Biafran forest refugia, inside curve of the Gulf of Guinea, from Cross River to Sanaga River (west Cameroon *sensu lato*). The late Holocene phase of dramatic climatic disturbance could also have been favourable for *C. canephora *diversification in Central Africa. Culminating about 2,500 years ago, it led to a catastrophic reduction in central African rainforests, in the region of south Cameroun, south Central African Republic, Gabon and Congo, and still exerts a major influence on the present forest vegetation distribution [53]. This event involved a brutal extension of savannas, favourable for the expansion of pioneering species such as oil palm [54]. The return of wetter conditions favourable for forest reinvasion began around 2,000 years B.P. from residual forests. This climatic disturbance caused fragmentation of the ancient Okoume (*Aucoumea klaineana*) distribution area into two subgroups, as suggested by the two tree varieties observed at the molecular level [21]. This process could also have produced the spatial genetic structuring of *C. canephora *in the Congolese zone. Both the intensity and length of these recent range expansion episodes could explain why coffee trees did not accumulate enough differentiation to lead to reproductive barriers and speciation.

#### Impact of human cultivation on inter-group gene flow and hybridization

The genetic structure analyses conducted in the present study seem to be efficient for reliable detection of admixtures and individual identification. Indeed, deviations from *a priori *diversity group classifications, are in line with issues concerning the introduction of non-indigenous wild or cultivated *C. canephora *plants or with the putative hybrid origin of cultivars.

Assignment tests confirmed that the landraces have remained genetically very close to the original wild populations. Indeed, since the 19^th ^century, many local cultivars were cultivated spontaneous forms of *C. canephora *directly collected in adjacent forest populations. This was the case in the Democratic Republic of the Congo, in Côte d'Ivoire (for "Kouilou" genotypes), Togo and Benin (for "Niaouli" genotypes), and Central African Republic (for "Nana" genotypes). Even improved material from INEAC (Table 2) has undergone few breeding cycles. Moreover, contact between local spontaneous forms and adjacent plantations could have promoted intermixing of individuals both in the wild and cultivated germplasm. In fact, according to the domestication syndrome definition (Hammer 1984), Robusta coffee trees should be considered as "cultivated" or "semi-domesticated" rather than "domesticated", since no heritable distinctive traits could be discerned when compared to their wild relatives.

The putative hybrid origin of some cultivars is suggested by the present data. In particular, most of the *a priori *group D (from Côte d'Ivoire and Guinea) and some of *a priori *group E (from southeast Cameroon and south Central African Republic) cultivated forms turned out to be putative F_1 _hybrids between these two genetic groups. Humans have contributed to this phenomenon since cultivated material originating from Central Africa, in particular from the Congo region, has been introduced in West African countries, such as in Côte d'Ivoire since 1930 (Portères, 1937). The low level of genetic introgression we observed in the putative hybrids suggests a recent contact between the local and exogenous material. Hybrids between the Guinean and Congolese group material had been previously identified by Berthaud [33] in cultivated variants on the basis of their vigour and yield. The use of intergroup hybrids and the efficiency of reciprocal recurrent selection was subsequently demonstrated [55]. This contrasts markedly with the cultivation history of *C. arabica*, whose varieties originated from a narrow genetic base [56]. Polymorphism was further reduced during selection cycles and by the predominant autogamy of *C. arabica*.

In this study, the putative intergroup hybrids, corresponding to various introgression levels detected and supposedly of wild origin, probably resulted from either culture escapes or from cross-pollinisation with neighbouring plantations.

Consequently, the core sets we propose contain both wild and cultivated accessions from the different groups, and a non-negligible fraction of putative hybrids. They provide good reference sets for further identification of spontaneous or cultivated material.

## Conclusion

The high degree of concordance between the SSR and RFLP data for diversity group differentiation indicates that our set of markers provided adequate genome coverage for accurate germplasm characterization. Even though these loci have likely evolved at different rates, they revealed comparable diversity structure with five well-differentiated groups corresponding to geographical patterning in the individuals. The molecular variation was correlated with the natural distribution in two postulated refugia areas, *i.e*. the Upper Guinea and Lower Guinea/Congo regions, separated by the Dahomey Gap.

A more intense and larger scale sampling would be required for more detailed geographical mapping of the diversity and more genetically precise refugia identification, especially in the Central African mosaic zone.

SSR genotyping provided highly informative data for multi-locus discrimination of individuals and putative hybrid detection. These data could be related to the recent history of coffee agricultural activities. Inter-group hybridizations were detected at a non-negligible frequency, especially between *a priori *group D ("Guinean" in West Africa) and E ("Congolese" in Central Africa). The previous observation of heterosis of Guinean-Congolese cultivars suggests that the high diversity included in these diversity groups represents an excellent genetic reservoir that could be tapped for adapted stock breeding schemes. This study also provides an excellent basis for determining the appropriate scale of wild population conservation and management.

## Methods

### Study species and sampled genotypes

*Coffea canephora *is a widespread species throughout west-central Africa [33]. More than 700 wild genotypes were collected by ORSTOM (now IRD, Institut de Recherche pour le Développement, France) in collaboration with CIRAD, FAO (Food and Agriculture Organization), IPGRI (International Plant Genetic Resources Institute), and MNHN (Muséum National d'Histoire Naturelle), between 1975 and 1987, in five African countries: Côte d'Ivoire and Guinea in West Africa; and Cameroon, Congo and Central African Republic in Central Africa [23-26,33]. These genotypes are conserved in the only reference collection for wild forms of *C. canephora*, *i.e*. the Divo collection, Côte d'Ivoire. In parallel, CIRAD assembled a collection of cultivated material, also conserved at the Divo experimental station. This collection contains more than 600 accessions of diverse origins: local varieties and populations, forms taken from village plantations, and selected material [26,33,35,57].

In this study, a total of 107 *Coffea canephora *genotypes (61 wild and 46 cultivated) were selected from the initial set analysed in Dussert et al. [35]. The wild genotypes were sampled in order to have a representation of each of the 40 forest populations studied throughout the range of the species (Figure 1, Table 1). For the cultivated material, a random proportional sampling was done for each of the 10 principal origins identified in collection (Table 2).

Total genomic DNA was extracted according to the method described by Ky et al. [58].

### RFLP analysis

Two restriction enzymes were used to digest the genomic DNA: *EcoR*I and *Hind*III. Out of the 26 initially tested probes, eight were retained for their polymorphic and mono-locus characteristics. Selected RFLP probes corresponded to previously mapped loci distributed on six linkage groups of the *C. canephora *linkage map (Table 3) [59].

### SSR analysis

Sixteen SSR loci (Table 3) were amplified as previously described [38,42] using a touchdown PCR profile optimised for each set of primers: touchdown 60°C to 55°C or touchdown 55°C to 50°C. PCR products were detected on an IR^2 ^Automated DNA Sequencer (LI-COR, model 4200L-2, Lincoln, NE, USA) using an M13 primer coupled to the infrared tag IRD700 or IRD800 after migration on 25 cm 6.5% KBplus (LI-COR, CAT# 827-05607) polyacrylamide gels. The gel images were processed by SAGA GT™ software (LICOR Biotech) to estimate the size of amplicons according to a 50–350 bp size standard (LI-COR, CAT# 829-05343, 829-05344).

The sixteen SSR loci were evenly distributed throughout the *Coffea *genome and nine of them mapped on nine different linkage groups of the intespecific map [*C. heterocalyx *× *C. canephora*) × *C. canephora*] (Table 3) [60].

### Data analysis

#### Genetic diversity

For each SSR and RFLP locus, we assessed genetic polymorphism within total or wild samples by calculating the observed number of alleles (*Na*), observed and expected heterozygosity (*H*_O _and *H*_E_), and the polymorphism information content (PIC) using the PowerMarker v3.25 software [61]. In these analyses, individual wild plants of possible hybrid origin, as determined by genetic structure analysis (see below), were excluded from the wild pool.

#### Genetic affinities among individual genotypes, Cluster analyses

Neighbour-joining trees were constructed using the shared-allele distances for both the RFLP and SSR data using PowerMarker v3.25 software [61]. Bootstrapping was conducted with 2000 replicates and the trees were implemented in the Phylip package [62] to obtain a consensus tree, visualized in TreeView (taxonomy.zoology.gla.ac.uk/rod/treeview.html). The Mantel matrix correspondence test was used to compare individual genetic distances generated by each marker type using PowerMarker v3.25.

In order to display patterns in the individual genetic distances, a principal coordinate analysis (PCO) was performed on both the RFLP and SSR dataset. For each individual, we calculated the frequencies of each allele (0, 0.5, and 1) at each locus, and used this data to perform a principal component analysis (PCA), "French PCA" in Statistica v6.1 . This analysis was computed for the whole sample, with cultivated and putative hybrids, as determined by genetic structure analysis (see below), included as additional passive elements.

#### Genetic structure

For statistical investigation of genetic structure of the *C. canephora *wild sample and detection of intermediate types (hybrids between diversity groups), two different Bayesian analyses were performed.

The genetic structure was first investigated for both RFLP and SSR datasets with the Bayesian approach in Structure v2.1[45,47]. Parameters were set at K = 5 for the number of groups, 30,000 for the burn-in time and 1,000,000 for the number of runs, with five repetitions. The number of clusters (K = 5) was confirmed as the value that maximized the increase in the posterior probability of the Ln *P*(D) data according to the formula [Ln *P*(D)_k_-Ln *P*(D)_k-1_], as suggested by Garnier et al. [63]. We assessed the proportion of qi membership of each genotype to the five genetic groups, *i.e*. the proportion of its genome drawn from each group. We assigned each individual genome to one group when the average proportion of membership was qi > 0.80, *i.e*. over 80% ancestry to their own cluster. In the case of admixed individuals, we jointly assigned them to two or more groups if the proportion of membership to each one was 0.20 < qi < 0.80. For each individual, we calculated a 90% CI of the qi parameter.

We also used the "leave one out" procedure and the method proposed by Cornuet et al. [64]. The Bayesian-based maximum likelihood test implemented in Geneclass2 v2.0 [46] has been shown to be effective in genotype assignment, even when populations deviate from Hardy-Weinberg equilibrium [65,66]. Each individual to be reassigned was removed from its source group and the frequency estimates of each locus were modified accordingly (Monte Carlo simulations of 1,000 independent individuals for each candidate group). Differences in log-likelihood values were computed to assign individuals to one group with a risk of 0.01. The reference groups used were the *a priori *A, B, C, D, and E groups. This software was also used to assign cultivated plants to the wild genetic groups.

#### Genetic differentiation

For the analysis of genetic differentiation within the wild sample, individual plants of possible hybrid origin were excluded. The partition of the genetic variation between wild genetic groups for both RFLP and SSR data was estimated with the *F*_ST _of Weir and Cockerham implemented in GENETIX [67]. Significance levels of pairwise *F*_ST _values were calculated using permutation tests (N = 1000). The correlation between the two *F*_ST _matrixes generated by each marker type was investigated by Mantel's test of matrix correspondence in GENETIX.

#### Core set

To assist in the use or conservation of wild and cultivated *Coffea canephora *germplasm, we defined core sets of accessions that capture the maximum RFLP or SSR diversity using the principal component score strategy (PCSS) [68]. Based on Khi-2 distances, a factorial analysis is applied to transform initial data into factor scores. Iterative selection of individuals maximising subset variability is based on their relative contribution to the generalised sum of squares (GSS), expressed in percentage.

## Authors' contributions

CG carried out the PCR amplification experiments and the genotyping, participated in the analyses and drafted the manuscript. SD obtained all the specimens, carried out the RFLP experiments and helped in analyzing the diversity data, and also drew up some of the figures. PH, SH and AK helped in analyzing the data. AdK and SH coordinated the project. VP served as the principal investigator of the project, participated in its design and coordination, helped in the data analysis, assisted in the drafting of the manuscript. All authors read and approved the final manuscript.
